# Night shift work, dietary patterns, and coronary heart disease

**DOI:** 10.1007/s10654-026-01362-w

**Published:** 2026-02-21

**Authors:** Diana A. Nôga, Elisa M. S. Meth, André P. Pacheco, Jonathan Cedernaes, Pei Xue, Christian Benedict

**Affiliations:** 1 Department of Pharmaceutical Biosciences, Uppsala University, Uppsala, Sweden; 2 Department of Research and Innovation, Division of Mental Health and Addiction, Oslo University Hospital, Oslo, Norway; 3 Institute of Clinical Medicine, Faculty of Medicine, University of Oslo, Oslo, Norway; 4 Department of Medical Sciences, Uppsala University, Uppsala, Sweden; 5 Department of Medical Cell Biology, Uppsala University, Uppsala, Sweden

**Keywords:** Night shift work, Ischemic heart disease, Dietary fiber, Meat consumption, Cardiovascular epidemiology, Prospective cohort study

## Abstract

Shift workers exhibit a higher incidence of coronary heart disease (CHD) than daytime workers. We examined whether this difference in CHD incidence between shift workers and daytime workers is associated with two dietary factors linked to lower CHD risk—daily fiber intake and meat avoidance—using UK Biobank data. The study included 222,801 participants (53.8% women; mean age 52.6 years), categorized as daytime workers, shift workers with no or occasional night shifts, or regular night shift workers. Dietary habits were assessed via touchscreen dietary questionnaires. During a median follow-up of 12.6 years, 12,265 fatal and non-fatal CHD events occurred. Compared with daytime workers, regular night shift work—but not shift work with no or occasional night shifts—was associated with higher CHD incidence (hazard ratio [HR] 1.10, 95% CI 1.01–1.20). HRs for CHD were higher (vs. daytime work) in both shift work groups among participants with lower daily fiber intake, whereas they were attenuated among those with higher fiber intake (p < 0.05 for interaction). Meat avoidance was associated with a 10.4% lower CHD HR compared with meat consumption (p = 0.020), although no significant interaction with work schedule was observed. In summary, while our finding that meat avoidance is associated with modestly lower CHD HR across work schedules aligns with established cardiovascular recommendations, our observation that higher fiber intake may reduce CHD differences specifically linked to night shift work highlights an additional, shift-specific dietary consideration that could complement the American Heart Association’s Life’s Essential 8.

**Supplementary Information:**

The online version contains supplementary material available at 10.1007/s10654-026-01362-w.

## Introduction

According to 2023 estimates, approximately 5% of people living in the European Union are employed in night shift work [1], while about five million workers in the United States perform this type of work [2]. Although night shift work is essential for societal services such as healthcare, firefighting, law enforcement, and transportation, it raises significant health concerns. By disrupting biological processes reserved for sleep, recovery, and restoration, night shift work can adversely affect overall health [3]. The cardiovascular system is particularly vulnerable to such circadian disruptions, with regular night shift work linked to an increased risk of cardiovascular diseases, especially coronary heart disease (CHD) [4]. Given the prevalence of night shift work and its well-established health risks, identifying effective and scalable interventions to mitigate CHD in this population is a pressing public health priority.

The American Heart Association (AHA) recognizes healthy dietary habits as a key component of the Life’s Essential 8 for cardiovascular health [5]. In this context, meta-analyses and large-scale studies suggest that fiber-rich diets, along with meat avoidance, may be particularly effective in promoting cardiovascular health [6–10]. However, it remains unclear whether the elevated incidence of CHD typically observed in night shift workers compared with daytime workers [4] is influenced by habitual fiber intake or meat avoidance. Understanding these relationships is critical for developing actionable dietary guidelines for this high-risk population. Therefore, using data from the UK Biobank, this study examined whether the elevated CHD hazard ratios (HRs) typically observed in regular night shift workers [4] are attenuated when their diet is characterized by high fiber intake and meat avoidance.

## Methods

### Study design and population

The UK Biobank is a prospective, population-based cohort of 502,366 individuals, recruited from the United Kingdom between 2006 and 2010. For the present study, data from 222,801 participants aged 38 to 71 years (53.8% women) from the UK Biobank baseline were used, with follow-up data available until September 30, 2021. Several criteria were applied to define the final cohort, including exclusion of participants who withdrew consent from the Biobank, were unemployed, had a diagnosis of CHD before or at the baseline assessment, or had missing exposure or covariate data, as shown in the online Supplemental Figure S1. All participants provided written informed consent, including consent for linkage to their medical records. The study was approved by the research ethics committee (11/NW/0382) and adhered to the principles of the Declaration of Helsinki.

### Classification of work schedule at baseline

Work schedule was determined for participants employed at baseline based on their responses to electronic questionnaire items: “Does your work involve shift work?” (referring to work outside traditional work hours) and “Does your work involve night shifts?” (referring to work during normal sleeping hours). Those who answered “never” or “rarely” to the shift work question were classified as daytime workers. Participants who reported shift work “sometimes,” “usually,” or “always” but indicated “never/rarely” or “sometimes” for night shifts were categorized as shift workers with no or occasional night shifts, including those who responded “sometimes” to the shift work question and “usually” or “always” to the night shift question. Those who answered “usually” or “always” to both questions were classified as regular night shift workers.

### Assessment of fiber and meat intake at baseline

In line with the methodology used in a previous UK Biobank study [11], daily fiber intake (in grams) was assessed through a series of questions about participants' consumption of fruits, vegetables, cereals, and bread, with additional details on the types of bread and cereal consumed. The questions included: “On average, how many heaping tablespoons of salad or raw vegetables do you eat per day? (Include lettuce and tomato in sandwiches; enter '0' if none)”; “On average, how many heaping tablespoons of cooked vegetables do you eat per day? (Do not include potatoes; enter '0' if none)”; “How many pieces of fresh fruit do you eat per day? (Count one apple, one banana, or 10 grapes as one piece; enter '0' if none)”; “How many pieces of dried fruit do you eat per day? (Count one prune, one dried apricot, or 10 raisins as one piece; enter '0' if none)”; “How many bowls of cereal do you eat per week?”; “What type of cereal do you mainly eat?”; “How many slices of bread do you eat each week?”; and “What type of bread do you mainly eat?”.

Weekly intake of processed meat was assessed based on responses to the touchscreen question: “How often do you eat processed meats (such as bacon, ham, sausages, meat pies, kebabs, burgers, or chicken nuggets)?” Unprocessed red meat intake was determined from responses to three separate questions: “How often do you eat pork? (Do not count processed meats such as bacon or ham)”; “How often do you eat lamb/mutton? (Do not count processed meats)”; and “How often do you eat beef? (Do not count processed meats).” For the analysis, participants were categorized based on their consumption of processed or unprocessed red meat, with those who reported consuming none (meat avoiders) compared with those who did (meat eaters).

### Identification and follow-up of CHD events

Fatal and non-fatal CHD events were identified based on the first occurrence of International Classification of Diseases, 10th Revision (ICD-10) codes “I20” to “I25” (UK Biobank identifiers [IDs]: 131296, 131298, 131300, 131302, 131304, 131306, respectively). These events were determined using data from primary care records, hospital inpatient records, death registries, and self-reported medical conditions. The study’s censoring date was September 30, 2021, based on the UK Biobank project’s approved data availability.

### Confounders

To ensure the robustness of the observed associations between work schedule, the dietary habits under study, and CHD HRs, we adjusted for a comprehensive set of factors. These included assessment center location, age, biological sex, ethnicity, socioeconomic status, education, smoking status, body mass index (BMI), average self-reported sleep duration, frequency of alcohol consumption, and self-reported chronotype. Both hypertension and type 2 diabetes are more prevalent among shift workers and are well-established CHD risk factors [12–18]. Accordingly, incident hypertension or type 2 diabetes—identified using ICD-10 codes E11, I10–I13, or I15—occurring prior to the first non-fatal or fatal CHD event was considered a confounder. Finally, given the well-established relationship between physical activity and cardiovascular health [19], we also adjusted for job-related physical demands (whether the job mainly involved walking or standing and whether it required heavy physical or manual labor) as well as weekly leisure-time physical activity, categorized as low, moderate, or high according to the International Physical Activity Questionnaire [20].

### Statistical analyses

All statistical analyses were conducted using R version 4.4.1 and SPSS version 30. Data are presented as mean ± standard deviation (SD), unless otherwise specified. Because 14.5% of participants (n = 32,197 of 222,801) had missing physical activity data, we applied multiple imputation using fully conditional specification (10 iterations with 5 imputed datasets). Predictors in the imputation model included smoking status, education, sex, age, and BMI. We also imputed 62 cases with missing alcohol consumption using the same procedure.

Cox proportional hazards regression was employed to estimate HRs and 95 percent confidence intervals (95% CIs). Time at risk, measured in years, was defined as the period from recruitment (2006–2010) to the first occurrence of a fatal or non-fatal CHD event, death, or the end of the follow-up period, whichever occurred first. Proportional hazards assumptions were verified using Kaplan–Meier survival curves. HRs and 95% CIs are reported as pooled estimates across the imputed datasets using Rubin’s rules [21].

Two Cox regression models were constructed for the analysis. In Model A, the association of each primary exposure—work schedule (categorical), daily fiber intake (grams), and meat avoidance (categorical)—with prospective CHD HRs was examined, adjusting for participants’ age and sex. Model B included all primary exposures for mutual adjustment, together with confounders (see previous section). To explore whether daily fiber intake or meat avoidance modifies the association between work schedule and CHD, multiplicative interactions were tested in Model B. Multiplicative interactions between sex and each primary exposure were also examined in Model B. Finally, other-cause death was considered a competing risk for CHD events, using Fine–Gray subdistribution hazards models implemented with the cmprsk package (version 2.2-12) in R. A p-value of less than 0.05 was considered statistically significant for both interaction terms and main effects.

## Results

Of the 222,801 participants, 83.6% were daytime workers, 13.1% were classified as shift workers with no or occasional night shifts, and 3.3% met the criteria for regular night shift work. Detailed cohort characteristics, stratified by work schedule, are presented in Table 1.

**Table 1:** Baseline characteristics, categorized by work schedule

Mean (SD)/N (%)	Daytime work	Shift work with no or occasional night shifts	Regular night shift work
	n = 186,221	n = 29,153	n = 7427
Age, years	52.8 (7.1)	51.9 (7.0)	51.2 (6.7)
Women	102,502 (55.0%)	14,304 (49.1%)	2995 (40.3%)
Ethnicity			
White	178,268 (95.7%)	26,667 (91.5%)	6680 (89.9%)
Asian	3769 (2.0%)	964 (3.3%)	263 (3.5%)
Caribbean or African	2622 (1.4%)	955 (3.3%)	331 (4.5%)
Others	1562 (0.8%)	567 (1.9%)	153 (2.1%)
Region of the assessment center			
England	164,842 (88.5%)	25,687 (88.1%)	6497 (87.5%)
Scotland	7749 (4.2%)	1213 (4.2%)	358 (4.8%)
Wales	13,630 (7.3%)	2253 (7.7%)	572 (7.7%)
Education			
No qualification	15,788 (8.5%)	3654 (12.5%)	1018 (13.7%)
Any other qualification	92,860 (49.9%)	18,184 (62.4%)	5245 (70.6%)
University degree	77,573 (41.7%)	7315 (25.1%)	1164 (15.7%)
Smoking status			
Never	109,489 (58.8%)	15,640 (53.6%)	3894 (52.4%)
Previous	59,264 (31.8%)	9335 (32.0%)	2275 (30.6%)
Current	17,468 (9.4%)	4178 (14.3%)	1258 (16.9%)
Townsend socioeconomic deprivation index^†^	− 1.6 (2.9)	− 0.7 (3.2)	− 0.7 (3.2)
Job mainly involves walking/standing			
Never/rarely	74,730 (40.1%)	4470 (15.3%)	945 (12.7%)
Sometimes	58,690 (31.5%)	8474 (29.1%)	1524 (20.5%)
Usually	24,592 (13.2%)	6077 (20.8%)	1786 (24.0%)
Always	28,209 (15.1%)	10,132 (34.8%)	3172 (42.7%)
Job involves heavy physical/manual work			
Never/rarely	134,965 (72.5%)	10,764 (36.9%)	1901 (25.6%)
Sometimes	33,112 (17.8%)	10,817 (37.1%)	2801 (37.7%)
Usually	9278 (5.0%)	3825 (13.1%)	1347 (18.1%)
Always	8866 (4.8%)	3747 (12.9%)	1378 (18.6%)
Physical activity level			
Low	33,075 (17.8%)	3559 (12.2%)	761 (10.2%)
Moderate	67,040 (36.0%)	8142 (27.9%)	1797 (24.2%)
High	60,372 (32.4%)	12,413 (42.6%)	3445 (46.4%)
Missing #	25,734 (13.8%)	5039 (17.3%)	1424 (19.2%)
Frequency of alcohol consumption			
Non-drinker	10,019 (5.4%)	2199 (7.5%)	630 (8.5%)
< 3 times/week	90,428 (48.6%)	15,719 (53.9%)	4316 (58.2%)
≥ 3 times/week	85,722 (46.0%)	11,229 (38.5%)	2477 (33.4%)
Missing #	52 (0.03%)	6 (0.02%)	4 (0.05%)
Sleep duration, hours/day	7.1 (0.9)	7.0 (1.0)	6.9 (1.1)
Chronotype			
Definitely morning	47,975 (25.8%)	7936 (27.2%)	1626 (21.9%)
More morning than evening	68,008 (36.5%)	9703 (33.3%)	2001 (26.9%)
More evening than morning	53,918 (29.0%)	8711 (29.9%)	2562 (34.5%)
Definitely evening	16,320 (8.8%)	2803 (9.6%)	1238 (16.7%)
Fiber intake, gram/day	14.2 (6.1)	14.0 (6.7)	13.9 (7.1)
Meat avoidance	11,726 (6.3%)	1709 (5.9%)	316 (4.3%)
BMI, kg/m^2^	27.0 (4.6)	27.9 (4.9)	28.3 (4.9)
History of T2D or hypertension prior to CHD event §	53,985 (29.0%)	9464 (32.5%)	2525 (34.0%)
Use of statins	14,977 (8.0%)	2581 (8.9%)	664 (8.9%)
Non-fatal and fatal CHD events occurring during follow-up	9922 (5.3%)	1806 (6.2%)	537 (7.2%)

*CHD* coronary heart disease (non-fatal and fatal), *BMI* body mass index, *T2D* type 2 diabetes

^†^The more positive, the worse the socioeconomic status [22]. ^#^Imputed for analysis, see *Statistical analyses* section. ^§^T2D or hypertension history was derived from registry records and considered present if diagnosed before the participant’s first CHD event

### Work schedule, dietary factors, and CHD outcomes

**Fig. 1:**
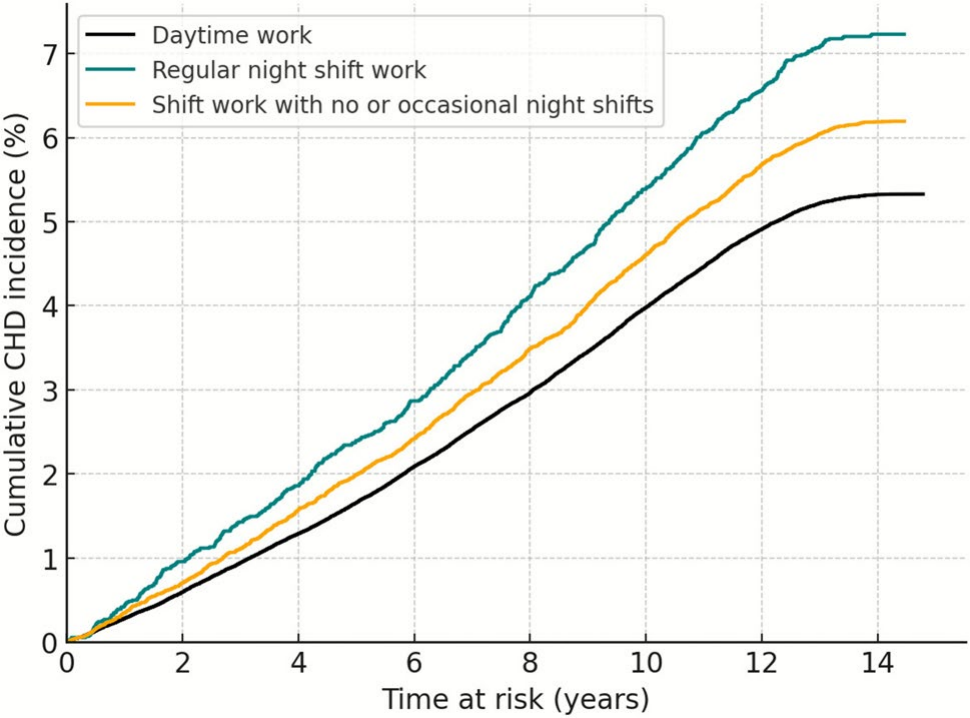
Cumulative incidence of CHD over 14 years of follow-up, stratified by work schedule. *CHD* coronary heart disease

The cohort accumulated a total of 2,722,510 person-years at risk over a median follow-up period of 12.6 years (25th percentile: 11.9 years; 75th percentile: 13.3 years), during which 12,265 individuals experienced CHD events (5.5% of the cohort). Figure 1 shows the relative number of incident CHD events (fatal and non-fatal) for each work schedule group, and Supplemental Table S1 presents the number of participants at risk during follow-up, stratified by work schedule. Compared with daytime workers, both shift workers with no or occasional night shifts and regular night shift workers had HRs [95% CIs] for incident CHD that were 1.203 [1.144 to 1.265] to 1.405 [1.288 to 1.533] times higher in Cox regression Model A (p < 0.001). In Cox regression Model B, only regular night shift workers showed a statistically significant elevation in the CHD HR compared with daytime workers (HR [95% CI], no or occasional night shifts vs. daytime workers: 1.019 [0.967 to 1.073], p = 0.488; regular night shift workers vs. daytime workers: 1.100 [1.006 to 1.203], p = 0.037).

**Fig. 2:**
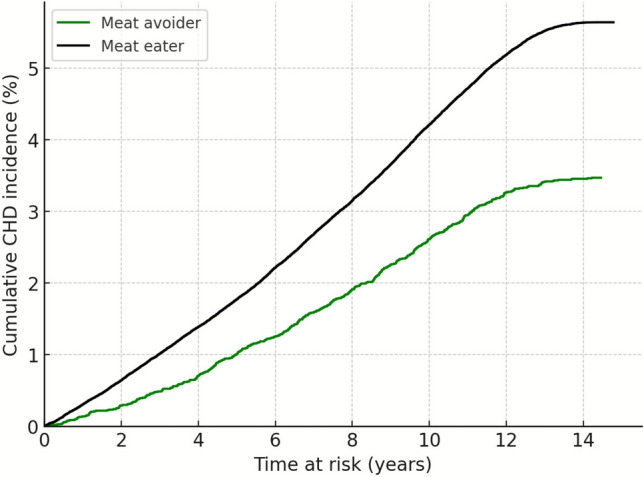
Cumulative incidence of CHD over 14 years of follow-up, stratified by meat consumption. *CHD* coronary heart disease

We next examined dietary factors. In Cox regression Model A, each additional gram of daily fiber intake was associated with an estimated 1.1% lower CHD HR (HR, 0.989; 95% CI 0.986 to 0.992; p < 0.001). In Model B, the corresponding change was 0.6% (HR, 0.994; 95% CI 0.991 to 0.997; p < 0.001). Furthermore, compared with meat eaters, meat avoiders had lower CHD HRs: 20.9% lower in Model A (HR, 0.791; 95% CI 0.722 to 0.867; p < 0.001) and 10.4% lower in Model B (HR, 0.896; 95% CI, 0.816 to 0.984; p = 0.022). Figure 2 illustrates incident CHD events (fatal and non-fatal) by meat consumption status, with Supplemental Table S2 reporting the number of participants at risk during follow-up for the same categories.

### Fine-gray analysis

Accounting for deaths from causes other than CHD as a competing event (n = 6907) yielded results consistent with those from Model B, the fully adjusted Cox regression model**.** Regular night shift workers had significantly higher sub-distribution hazards of incident CHD compared with daytime workers (sub-distribution HR, 1.098; 95% CI 1.004 to 1.201; p = 0.042), whereas shift workers with no or occasional night shifts did not differ significantly from daytime workers (1.017; 95% CI 0.965 to 1.072; p = 0.523). Each additional gram of daily fiber intake was associated with a lower sub-distribution hazard of CHD (0.995; 95% CI 0.992 to 0.998; p < 0.001). Finally, compared with meat eaters, meat avoiders had lower sub-distribution hazards of CHD during follow-up (0.894; 95% CI 0.814 to 0.982; p = 0.019).

### Interactions between work schedule, diet, and CHD

Using daytime workers as the reference group, the association between shift work and incident CHD did not vary according to participants’ meat consumption status. The pooled interaction terms for shift work × meat consumption status (using meat eaters as the reference) were not statistically significant (shift work with no or occasional night shifts × meat avoidance: β [SE] = − 0.098 [0.137], p = 0.475; regular night shifts × meat avoidance: β [SE] = − 0.165 [0.296], p = 0.577), indicating that meat avoidance does not appear to modify the association between shift work and CHD HRs.

**Fig. 3:**
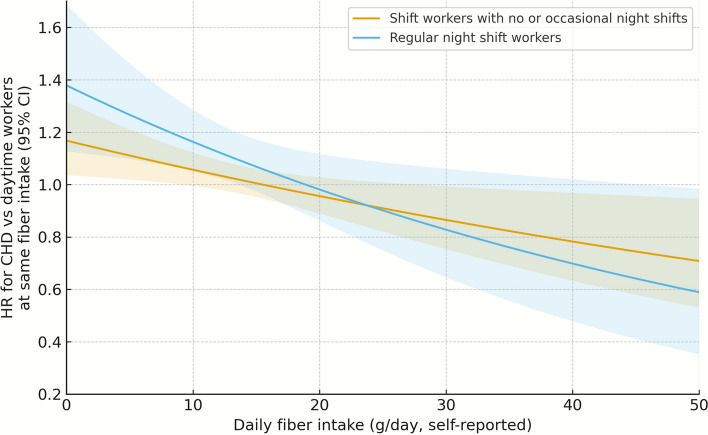
Adjusted hazard ratios for incident CHD in shift workers according to self-reported daily fiber intake. Curves show hazard ratios for shift workers with no or occasional night shifts and regular night shift workers, each compared with daytime workers at the same level of fiber intake. Shaded areas represent 95% confidence intervals, based on the variance–covariance matrix of the interaction model. Models were fitted in the multiply imputed cohort and adjusted for age, sex, BMI, socioeconomic status, assessment center, chronotype, ethnicity, education, smoking, sleep duration, physical activity, work-related physical demands, meat and alcohol intake, statin use, and prevalent diabetes and/or hypertension. *HR* hazard ratio, *CHD* coronary heart disease

A significant multiplicative interaction was observed between daily fiber intake (grams) and work schedule. This interaction was significant for both shift work with no or occasional night shifts (β [SE] = − 0.010 [0.004], p = 0.011) and regular night shifts (β [SE] = − 0.016 [0.007], p = 0.020). Based on these interactions, the estimated CHD HR relative to daytime workers reaches approximately 1 at around 15 g/day of fiber for shift workers with no or occasional night shifts and around 19 g/day for regular night shift workers (Fig. 3). At fiber intakes above these thresholds, the model predicts CHD HRs slightly below 1. This pattern should be interpreted as a consequence of the statistical interaction in the model rather than a causal protective effect of shift work itself; similarly, the elevated HRs at very low fiber intakes reflect the interaction structure rather than a definitive increase in CHD hazard. Overall, these results underscore that higher daily fiber intake is associated with attenuation of CHD HRs among shift workers, highlighting the potential cardiovascular benefit of a fiber-rich diet in this population.

In Cox regression Model B, no significant multiplicative interactions were observed between sex (reference: women) and any of the primary exposures: sex × shift work with no or occasional night shifts (β [SE] = − 0.108 [0.056], p = 0.053), sex × regular night shifts (β [SE] = − 0.109 [0.102], p = 0.284), sex × daily fiber intake (β [SE] = 0.002 [0.003], p = 0.586), or sex × meat avoidance (β [SE] = − 0.059 [0.094], p = 0.532). Sex-specific CHD HRs and 95% CIs for work schedule, fiber intake, and meat avoidance are provided in Supplemental Table S3.

## Discussion

A fiber-rich diet and meat avoidance have previously been associated with lower CHD incidence [6–10]. Whether these associations extend to night shift workers—who already exhibit higher CHD HRs [4]—has been less well understood. In this large prospective cohort of middle-aged adults, we observed a significant interaction between self-reported daily fiber intake and work schedule. Specifically, CHD HRs among night shift–working middle-aged adults were higher at lower fiber intakes, with the model indicating that HRs approached those of daytime workers at moderate fiber intake levels, approximately 15–19 g/day in this cohort. These intake values are cohort-specific and may not directly extrapolate to populations with different characteristics, such as age or ethnicity. Overall, the findings indicate that higher daily fiber intake may help attenuate the elevated CHD HRs associated with night shift work, although the observational nature of this study limits causal inference.

Higher fiber intake may be linked to lower CHD HRs through several biological pathways, including improvements in blood lipid profiles, weight management, glycemic control, gut microbiota composition, and reductions in systemic inflammation [23–26]. Each of these factors plays an important role in long-term cardiovascular health [27–31]. The stronger attenuation of CHD HRs observed among shift workers may reflect the additional cardiovascular stress imposed by night work—such as circadian disruption, elevated inflammation, and higher blood pressure [32]—which could make the benefits of fiber more apparent. By contrast, daytime workers had more favorable baseline health characteristics—e.g., lower BMI and lower prevalence of type 2 diabetes and hypertension (Table 1). Under the assumption that baseline risk influences detectability of dietary associations, these profiles may have offered less opportunity for fiber-related differences to appear in cardiovascular outcomes.

In contrast to the interaction observed with fiber intake, lower CHD HRs were observed among individuals who avoided meat, regardless of work schedule. This aligns with prior research showing that low meat consumption or vegetarian dietary patterns are associated with improved metabolic profiles relevant to long-term cardiovascular health, including lower incidence of type 2 diabetes and hypertension [33, 34]. Importantly, the associations between meat avoidance and CHD HRs in the present study were independent of daily fiber intake, as both exposures were mutually adjusted in the analyses, suggesting that the potential long-term cardiovascular benefits of meat avoidance are not primarily driven by higher fiber consumption (e.g., through increased intake of plant-based foods). Mechanistically, meat avoidance is associated with lower intake of saturated fat and heme iron, improvements in blood lipid profiles, reductions in systemic inflammation, and a healthier gut microbiota composition [35–37], each of which is known to support cardiovascular health [27, 30, 38, 39].

### Critical points in interpretation

First, although our analyses suggest that daily fiber intake may modify CHD HRs in relation to work schedule, these results do not allow conclusions about whether other cardioprotective dietary factors (e.g., omega-3 fatty acid–rich diets) would have similar interaction effects. Moreover, while meat avoidance was associated with lower CHD HRs regardless of work schedule, it is important to recognize that moderate meat consumption provides meaningful amounts of protein, iron, vitamin B12, and other essential nutrients. Therefore, dietary recommendations require careful individual consideration, and these findings should not be interpreted as advocating universal meat avoidance.

Second, daily fiber and meat intake were assessed only at baseline, which introduces uncertainty regarding long-term dietary habits over the follow-up period. However, reproducibility analyses in a UK Biobank subsample suggest that baseline dietary measures adequately rank individuals by relative intake over time [11].

Third, although we adjusted for a comprehensive set of confounders—including socioeconomic status, smoking, BMI, alcohol consumption, physical activity, sleep duration, and chronotype—residual confounding from unmeasured lifestyle behaviors cannot be ruled out. For example, nighttime eating, which was not assessed in the present study, has been associated with cardiovascular outcomes in experimental studies [40]. Furthermore, a more nuanced assessment of smoking habits or body adiposity could have improved our model’s ability to account for their potential influence on CHD HRs.

Fourth, our analyses included incident hypertension and type 2 diabetes occurring prior to CHD onset; however, some individuals may have undiagnosed conditions. In UK Biobank, these diagnoses are captured through hospital episodes, primary care records, and self-report, reducing but not eliminating the risk of misclassification. Any remaining misclassification is likely non-differential with respect to work schedule and thus would bias our estimates toward the null rather than inflate associations.

Fifth, although higher fiber intake may offer additional cardioprotective benefits for night shift workers, caution is warranted in interpretation and in public health messaging. High fiber intake may not be appropriate for individuals with certain gastrointestinal conditions [41]. Likewise, while a fiber-rich diet may confer added benefit, it should be viewed as a complement—not a substitute—for established cardioprotective behaviors. Traditional cardiovascular health behaviors and risk factors—such as regular physical activity, smoking avoidance, healthy sleep, and optimal blood pressure—remain key for reducing CHD HRs [5], including in shift-working populations.

Finally, the UK Biobank cohort consists primarily of middle-aged adults of White ethnicity, which may limit generalizability to more diverse populations [42]. Future longitudinal studies with repeated dietary assessments, broader population representation, and detailed cardiometabolic phenotyping are needed to verify and extend these observations.

## Conclusions

Our findings indicate that specific dietary modifications may influence CHD HRs among shift workers. Meat avoidance was consistently associated with lower CHD HRs across all work schedules. Additionally, higher daily fiber intake appeared to offset the elevated long-term CHD HRs typically seen in night shift workers. These results suggest that reducing meat consumption may benefit cardiovascular health in the general workforce, while increasing fiber intake—when not medically contraindicated—may help mitigate CHD HRs among night shift workers. Overall, our study adds nuance to the American Heart Association’s Life’s Essential 8 [5] by highlighting how particular dietary choices can further support cardiovascular health. Future studies with repeated dietary assessments, more diverse populations, and detailed cardiometabolic phenotyping are needed to confirm these findings and establish the long-term impact of diet on cardiovascular outcomes in shift-working populations.

## Supplementary Information

Below is the link to the electronic supplementary material.

Supplementary file1 (DOCX 405 KB)
