# Supplementary material for: Night shift work, dietary patterns, and coronary heart disease
Source: Eur J Epidemiol. 2026 Feb 21;41(4):435–43. doi: 10.1007/s10654-026-01362-w (PMC13331945; doi:10.1007/s10654-026-01362-w)
Supplement: Supplementary file 1 — Supplementary file1 (DOCX 405 KB) [file 10654_2026_1362_MOESM1_ESM.docx]

**- Online Supplement -**

**Night Shift Work, Dietary Patterns, and Coronary Heart Disease**

*European Journal of Epidemiology*

Diana A. Nôga^1^, Elisa M. S. Meth^1^, André P. Pacheco^1,2,3^, Jonathan Cedernaes^4,5^,

Pei Xue^1^, Christian Benedict^1^

**^1^** Department of Pharmaceutical Biosciences, Uppsala University, Uppsala, Sweden

**^2^** Department of Research and Innovation, Division of Mental Health and Addiction, Oslo University Hospital, Oslo, Norway

**^3^** Institute of Clinical Medicine, Faculty of Medicine, University of Oslo, Oslo, Norway

**^4^** Department of Medical Sciences, Uppsala University, Uppsala, Sweden

**^5^** Department of Medical Cell Biology, Uppsala University, Uppsala, Sweden

**Correspondence to:** Diana Nôga (diana.noga.morais@uu.se) or Christian Benedict (christian.benedict@uu.se).

**Fig. S1** Final sample estimation


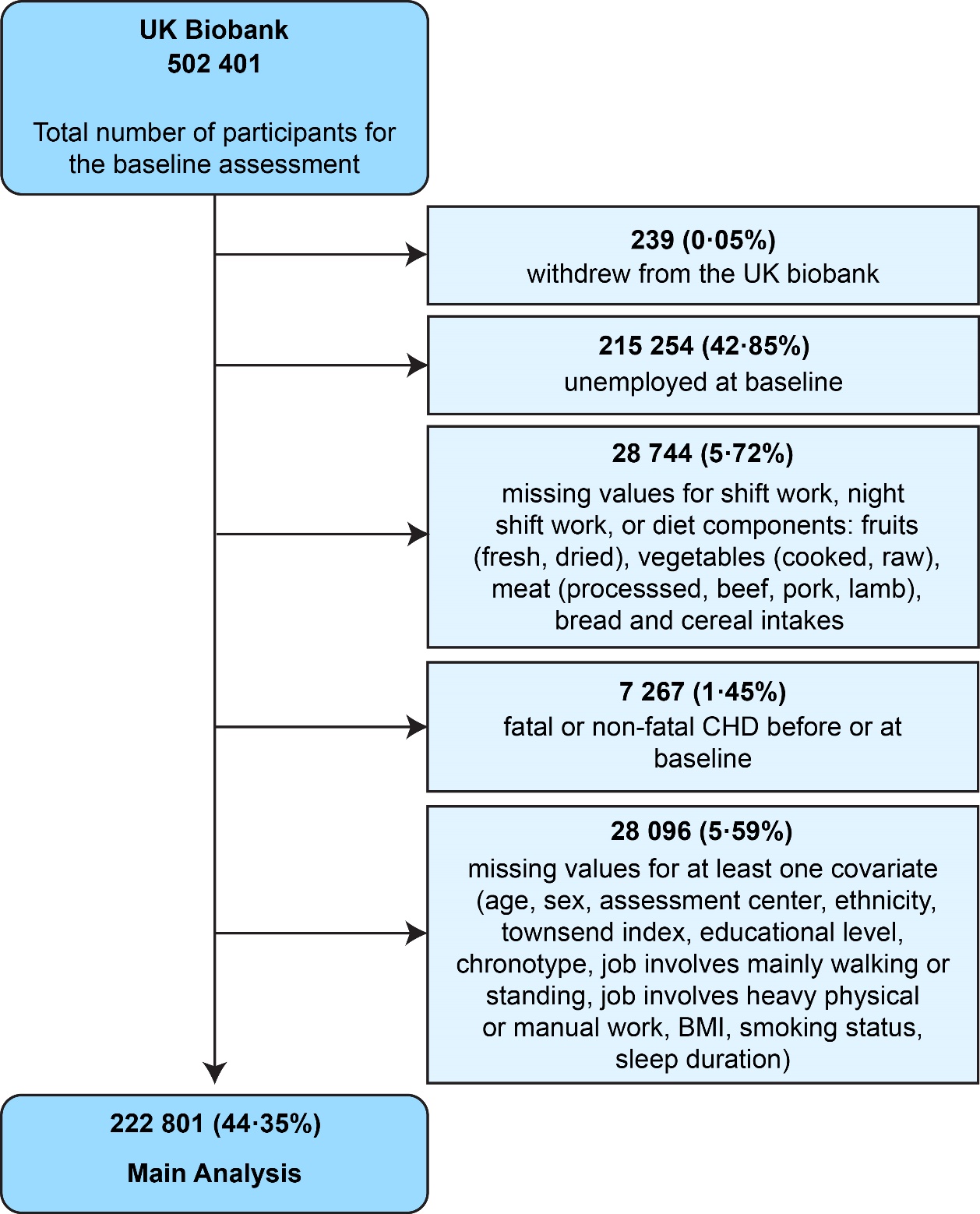


**Table S1** Number of participants at risk, categorized by work schedule

| Exposure group | No. at risk | | | | | | | |
| --- | --- | --- | --- | --- | --- | --- | --- | --- |
|  | Baseline | <2 years^#^ | <4 years^#^ | <6 years^#^ | <8 years^#^ | <10 years^#^ | <12 years^#^ | <14 years^#^ |
| Daytime work | 186221 | 184733 | 182772 | 180485 | 177947 | 174959 | 129651 | 9088 |
| Shift work with no or occasional night shifts | 29153 | 28883 | 28520 | 28151 | 27681 | 27175 | 19729 | 1337 |
| Regular night shift work | 7427 | 7335 | 7245 | 7127 | 6995 | 6867 | 5084 | 316 |

^#^ Time since baseline (2006-2010)

**Table S2** Number of participants at risk, categorized by meat consumption status

| Exposure group | No. at risk | | | | | | | |
| --- | --- | --- | --- | --- | --- | --- | --- | --- |
|  | Baseline | <2 years^#^ | <4 years^#^ | <6 years^#^ | <8 years^#^ | <10 years^#^ | <12 years^#^ | <14 years^#^ |
| Meat eater | 209050 | 207264 | 204955 | 202304 | 199309 | 195843 | 145019 | 9989 |
| Meat avoider | 13751 | 13688 | 13582 | 13459 | 13314 | 13158 | 9445 | 752 |

^#^ Time since baseline (2006-2010)

**Table S3** Sex-specific Cox regression estimates

| Exposure of interest | Female subgroup |  | Male subgroup |  |
| --- | --- | --- | --- | --- |
|  | Model A, HR [95% CI] | Model B, HR [95% CI] | Model A, HR [95% CI] | Model B, HR [95% CI] |
| Daytime workers | 1 | 1 | 1 | 1 |
| Shift workers with no or occasional night shifts | 1.299 [1.187 to 1.422] | 1.056 [0.960 to 1.162] | 1.159 [1.091 to 1.231] | 0.998 [0.938 to 1.063] |
| Regular night shift workers | 1.600 [1.347 to 1.901] | 1.129 [0.944 to 1.350] | 1.334 [1.206 to 1.476] | 1.088 [0.981 to 1.206] |
|  |  |  |  |  |
| Fiber intake, gram per day | 0.984 [0.978 to 0.990] | 0.993 [0.987 to 0.998] | 0.990 [0.987 to 0.994 | 0.995 [0.991 to 0.998] |
|  |  |  |  |  |
| Meat eater | 1 | 1 | 1 | 1 |
| Meat avoider | 0.829 [0.729 to 0.944] | 0.970 [0.850 to 1.107] | 0.758 [0.665 to 0.863] | 0.848 [0.742 to 0.968] |

Values are presented as hazard ratios (HRs) with 95% confidence intervals (95% CIs). Models were fitted using the multiply imputed cohort. Model A included the exposure of interest and age. Model B included all exposures of interest simultaneously for mutual adjustment, as well as age, BMI, socioeconomic status, assessment center, chronotype, ethnicity, education, smoking, sleep duration, physical activity, work-related physical demands, meat and alcohol intake, statin use, and prevalent diabetes and/or hypertension.
